# Neighborhood Ethnic Diversity and Behavioral and Emotional Problems in 3 Year Olds: Results from the Generation R Study

**DOI:** 10.1371/journal.pone.0070070

**Published:** 2013-08-14

**Authors:** Ilse J. E. Flink, Rick G. Prins, Johan J. P. Mackenbach, Vincent W. Jaddoe, Albert Hofman, Frank C. Verhulst, Henning Tiemeier, Hein Raat

**Affiliations:** 1 The Generation R Study Group, Erasmus University Medical Centre, Rotterdam, The Netherlands; 2 Department of Public Health, Erasmus University Medical Centre, Rotterdam, The Netherlands; 3 Department of Epidemiology, Erasmus University Medical Centre, Rotterdam, The Netherlands; 4 Department of Pediatrics, Erasmus University Medical Centre, Rotterdam, The Netherlands; 5 Department of Child and Adolescent Psychiatry, Erasmus University Medical Centre, Rotterdam, The Netherlands; The University of Queensland, Australia

## Abstract

**Background:**

Studies suggest that neighborhood ethnic diversity may be important when it comes to understanding ethnic inequalities in mental health. The primary aim of this study was to investigate whether neighborhood ethnic diversity moderated the association between the ethnic minority status and child behavioral and emotional problems.

**Methods:**

We included 3076 preschoolers participating in the Generation R Study, a birth cohort study in Rotterdam, the Netherlands. At child age 3-years, parents completed the Child Behavior Checklist (CBCL/1,5-5). Individual-level data, assessed with questionnaires, was combined with neighborhood-level data. Multi-level logistic regression models predicted the Odds Ratios for the CBCL total problems score as a function of maternal ethnic background and neighborhood ethnic diversity, computed with the Racial Diversity Index and categorized into tertiles. Interaction on the additive scale was assessed using Relative Access Risk due to Interaction.

**Results:**

Being from an ethnic minority was associated with child behavioral and emotional problems in unadjusted (OR 2.76, 95% CI 1.88–4.04) and adjusted models (OR 2.64, 95% CI 1.79–3.92). Residing in a high diversity neighborhood was associated with child behavioral and emotional problems in unadjusted (OR 2.03, 95% CI 1.13–3.64) but not in adjusted models (OR 0.89, 95% CI 0.51–1.57). When stratifying by the three levels of neighborhood ethnic diversity, ethnic inequalities in behavioral and emotional problems were greatest in low diversity neighborhoods (OR 5.24, 95%CI 2.47–11.14), smaller in high diversity neighborhoods (OR 3.15, 95% CI 1.66–5.99) and smallest in medium diversity neighborhoods (OR 1.59, 95% CI 0.90–2.82). Tests for interaction (when comparing medium to low diversity neighborhoods) trended towards negative on both the additive and multiplicative scale for the maternal-report (RERI: −3.22, 95% CI −0.70–0.59; Ratio of ORs: 0.30, 95% CI 0.12–0.76).

**Conclusion:**

This study suggests that ethnic inequalities in child behavioral and emotional problems may be greatest in ethnically homogeneous neighborhoods.

## Introduction

Previous studies have shown that differences in behavioral and emotional problems (e.g. attention problems) between ethnic minority and majority children can already be detected in the preschool years [1]. Understanding which factors contribute to these ethnic differences is of importance for the prevention and/or early detection of behavioral and emotional problems in minority children.

Ecological models postulate that the neighborhoods in which children grow up influences their health and wellbeing [2]. In a recent review on the significance of neighborhood context for child and adolescent health, Sellstrom and Bremberg [3] found that after controlling for individual characteristics, neighborhood socio-economic status and social climate had an impact, albeit small and moderate, on birth weight, injuries, behavioral problems, and child maltreatment.

One of the neighborhood factors which can exert an influence on child health and development is neighborhood ethnic diversity [4]–[8]. For instance, Hurtado [6] showed that growing up and interacting with peers in ethnically diverse settings led to more positive cognitive, social and democratic outcomes in youth. Seaton et al. [5] showed that residing in a neighborhood with a medium level of ethnic diversity buffered the negative association between racism and adolescent self-esteem.

Studies have suggested that ethnic background and the level of neighborhood ethnic diversity may have a combined effect on mental health [5], [9]–[11]. For instance, in a Dutch study, Gieling et al [9] found a negative association between school ethnic diversity, defined by the percentage of ethnic minority pupils, and externalizing problems, defined by “conflicts with other people and expectations for children's behavior” [12], however, only for ethnic minority adolescents. Although it is unclear which level of ethnic diversity is most beneficial for the health of ethnic minorities, possibly due to different definitions of ethnic diversity and/or the use of different cut-offs, there is an overall agreement that the health of ethnic minorities is worst off in ethnically homogeneous settings with a relatively large percentage of the majority group [4]. The main explanation for this has been that in these settings, ethnic minority groups are made more aware of their minority status [13]. As a result, racist and prejudiced attitudes and beliefs may be more common and this in turn impacts the mental health of ethnic minorities [4]. In settings which are more ethnically diverse, interactions between different ethnic groups may lead to more social integration and connectedness [13] and perceptions of safety [14] which enhances the mental health of ethnic minorities.

Although previous studies have shown that there are ethnic inequalities in child mental health [1], [15] there are, to our knowledge, no studies that have investigated whether this association depends on the level of neighborhood ethnic diversity. Therefore, the primary aim of this study was to investigate whether neighborhood ethnic diversity moderated the association between the ethnic minority status and child behavioral and emotional problems. Because it is unclear which level of neighborhood ethnic diversity may be most beneficial for the mental health of ethnic minority preschoolers, this study was explorative in nature.

## Methods

### Participants

This study was embedded in the Generation R study, a prospective population-based cohort from fetal life onwards in Rotterdam, the Netherlands [16]. In short, mothers were eligible to participate if they were resident in Rotterdam during their delivery date (April 2002 till January 2006). Midwives and obstetricians informed and invited eligible mothers to participate during their first prenatal visit in routine care [16]. The Medical Ethics Committee of the Erasmus University Medical Center in Rotterdam approved this study. Written informed consent was obtained from all participants.

**Figure 1:**
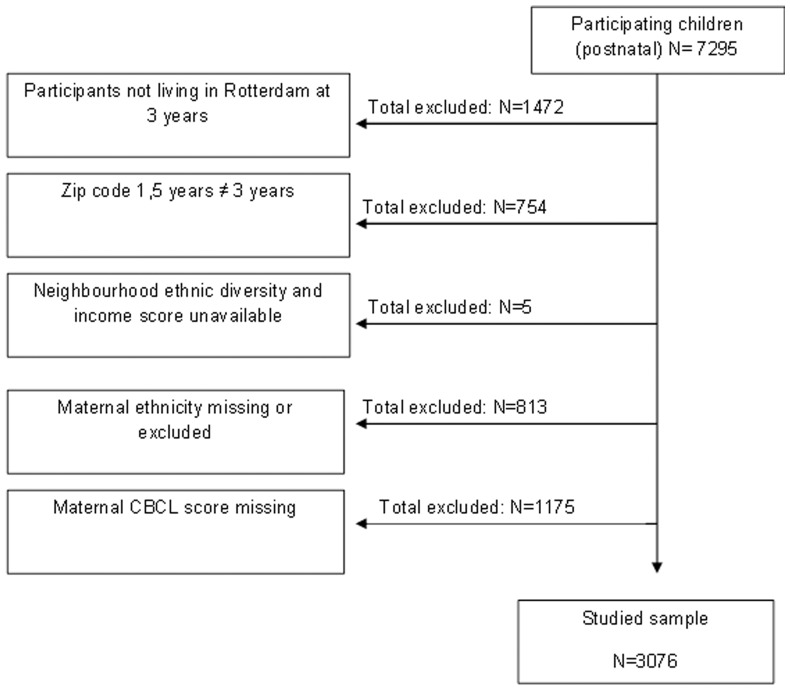
Flowchart of the study population.

Full consent for the postnatal phase was obtained from 7295 participants. Children who did not live in Rotterdam at age 3 years (N = 1472) were excluded. To make sure that the children included in this study were living in the neighborhood for a sufficient amount of time, we also excluded children who did not live in the same postal code area for at least 1.5 years (N = 754). Further excluded were those living in neighborhoods with no ethnic diversity and income score (n = 5) and children for whom maternal ethnic background was missing and whose mothers were from smaller or heterogeneous ethnic minority groups (N = 813). Lastly, children with no maternal CBCL score (N = 1175) were excluded leaving 3076 children for analysis (see figure 1).

### Measures

Data for this study were retrieved from medical and municipal records, and collected by prenatal and postnatal questionnaires.

#### Behavioral and emotional problems

Mothers and fathers were asked to fill out the Child Behavior Checklist (CBCL/1,5-5) separately when the child was 3 years old. The CBCL/1,5-5, is a parent-report questionnaire that contains 99 problem items rated on a 3-point scale: 0 (not true), 1 (somewhat or sometimes true) and 2 (very true or often true). In this study we used the Total problems score which is the sum of the scores of the 99 problem items. Good reliability and validity have been reported for the CBCL/1,5-5 [17]. Internal validity of the maternal-report of the Total Problems scale in this population was α = 0.93. We chose to present *maternal*-reported Total problems as main findings because research has shown that mothers are usually more reliable informants when it comes to assessing the health of their children [18], [19]. Additionally, more mothers than fathers completed the CBCL at 36 months. Findings for paternal-reported behavioral and emotional problems were however included as supplementary material.

#### Maternal ethnic background

We classified the children in this study according to maternal ethnic background because mothers play an important role in young children's lives and their ethnic background and experiences of, amongst others; acculturation and discrimination are most likely to influence child behavioral and emotional problems [20], [21]. Maternal ethnic background was determined by the country of birth of the mother and the mother's parents, a classification employed by Statistics Netherlands [22]. If the mother or one of her parents was born outside the Netherlands, this country of birth determined the ethnic background. If both parents were born outside the Netherlands, the country of birth of the mother's mother determined the ethnic background. Women with a Surinamese background were further classified as Surinamese Hindu or Surinamese Creole. Subgroups included in the study were: Dutch (N = 2149), Other European (N = 273), Antillean (N = 53), Cape Verdean (N = 74), Surinamese Hindu (N = 66), Surinamese Creole (N = 60), Moroccan (N = 135) and Turkish (N = 266); which are considered the largest ethnic minority groups in Rotterdam [23]. As individual ethnic subgroups were too small to address a cross-level interaction between neighborhood ethnic diversity and maternal ethnic background we grouped the ethnic subgroups into Dutch (N = 2149) and non-Dutch/ethnic minority (N = 927). As a sensitivity analysis, we also considered paternal ethnic background for which a similar classification was employed.

#### Neighborhood ethnic diversity

As suggested by Budescu and Budescu [4], the measure of ethnic diversity should be “sensitive to the relative proportion of each ethnic or racial group to the overall composition in a particular context”. Hence, we defined neighborhood ethnic diversity using the Racial Diversity Index which captures both the number of ethnic groups in the neighborhood as well as the relative representation of these groups [5], [14]. The index was computed using the following formula:
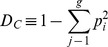
In the formula, *D_C_* represents the level of neighborhood ethnic diversity and p*_i_* the proportion of residents in the neighborhood who belong to ethnic group *i*. The p*_i_* is then summed across *g* groups in the neighborhood. A higher value on the index represents higher ethnic diversity. For instance, in a neighborhood (i.e. Blijdorpsepolder) where 7.1% are Antillean, 92.9% are Dutch the diversity score is 0.13. In contrast, in another neighborhood (i.e. Agniesebuurt) where 13.3% are Surinamese, 2,8% Antillean, 4.0% Cape Verdean, 15% Turkish, 10.7% Moroccans, 35.4% Dutch, 8.1% other non-Western, 5.5% other European and 5.2% other Western the diversity score is 0.81.

Data on ethnic composition of the neighborhoods was provided at the zip code level by the Rotterdam Centre for Statistics [23]. In our study, ethnic diversity of neighborhoods ranged from 0.06 to 0.85. Because it has been suggested that the relationship between neighborhood ethnic diversity and health outcomes may not be linear [4], [5], we recoded the continuous measure of neighborhood ethnic diversity into tertiles which is in line with other studies [11], [24]. The first category was considered low diversity and ranged from 0.06 to 0.43. The second category was considered medium diversity and ranged from 0.44 to 0.66. The third category was considered high diversity and ranged from 0.67 to 0.85.

#### Individual level confounders

The following individual-level factors were treated as potential confounders: gender and age of the child, maternal age, marital status (married/cohabiting or no partner); parity, maternal education, classified as ‘low’ (primary school, lower vocational training, intermediate general school, 3 years general secondary school), ‘medium’ (>3 years general secondary school; intermediate vocational training; 1st year higher vocational training), and ‘high’ (higher vocational training, Bachelor's degree, higher academic education and PhD); monthly net household income, classified as ‘<1200 €’ (below social security level), ‘1200–2000 €’ and ‘>2000 €’ (more than modal income).

#### Neighborhood level confounders

Adjustment for neighborhood wealth is useful to mitigate area-level confounding [25]. Hence, neighborhood wealth, determined by the average yearly household income per zip code, was considered a potential confounder. The measure of neighborhood wealth was provided by the Rotterdam Centre for Statistics [23]. Additionally we included the degree of urbanity as a potential confounder. Degree of urbanity was measured on a zip code level and was retrieved from Statistics Netherlands [26]. The measure was based on the number of addresses per km^2^ (1 = urban: more than 2499 addresses/km^2^; 2 = semi-urban: 1500–2499 addresses/km^2^; 3 = intermediate urban-rural: 1000–1499 addresses/km^2^; 4 = semi-rural: 500–999 addresses/km^2^; and 5 = rural: up to 499 addresses per km^2^).The urbanity index ranges from 1 to 5, with 1 including the most urban areas and 5 including the most rural areas.

### Statistical analyses

To handle missing data in the individual-level confounders, multiple imputation was applied [27]. Ten imputed datasets were generated using a fully conditional specified model, thus taking into account the uncertainty of the imputed values. Imputations were based on the relationship between all the individual-level variables included in this study.

Frequency tables and cross tabulations were used to explore characteristics of the study population (table 1). Because the CBCL Total Problems scores were skewed and could not be normalized, we dichotomized the scores according to the 83rd percentile borderline cut-offs of a Dutch reference population [28]. We used multi-level logistic regression with random effects to test the association between neighborhood ethnic diversity and CBCL total problems (table 2). We estimated an empty model first to determine the clustering of behavioral and emotional problems within neighborhoods. Model 1 is the association between maternal ethnic background and CBCL total problems score adjusted for individual level confounders. Model 2 is the association between neighborhood ethnic diversity and CBCL total problems score adjusted for neighborhood level confounders. The last model, Model 3, is the fully adjusted model including all individual- and neighborhood-level covariates.

**Table 1:** Characteristics of the population (N = 3076).

		*N (%)*	*Range*	*Mean (SD)*
**Child characteristics**				
Gender		2998		
	Boys	1506 (50.2)		
	Missing	78 (2.5)		
Age during questionnaire (months)		3076	34.0–51.2	36.6 (1.3)
CBCL total problems		3076		
	Above cut-off	196 (6.4)		
**Maternal/family characteristics**				
Age at intake (years)		3076	16.2–46.3	31.8 (4.6)
Ethnic background		3076		
*Dutch*		2149 (69.9)		
*Non-Dutch (ethnic minorities)*		927 (30.1)		
	Other European	273 (8.9)		
	Antillean	53 (1.7)		
	Cape Verdean	74 (2.4)		
	Moroccan	135 (4.4)		
	Surinamese Creole	60 (2.0)		
	Surinamese Hindu	66 (2.1)		
	Turkish	266 (8.6)		
Educational level		2973		
	High	1653 (55.6)		
	Mid	1118 (37.6)		
	Low	202 (6.8)		
	Missing	103 (3.3)		
Income		2490		
	>2000	1793 (72.0)		
	1200–2000	436 (17.5)		
	<1200	261 (10.5)		
	Missing	586 (19.1)		
Marital status		2963		
	Single	229 (7.7)		
	Missing	113 (3.7)		
Parity		2973		
	Nulli	1596 (53.7)		
	Missing	103 (3.3)		
**Neighborhood characteristics**				
Neighborhood income (×1000 Euros)		3076	21.4–62.9	34.2 (10.6)
Level of urbanity		3076	1–5	1.51 (0.91)
Neighborhood ethnic diversity		3076		
	Low diversity	988 (33.3)		
	Medium diversity	1093 (35.5)		
	High diversity	995 (32.1)		

Values are percentages for categorical variables, means (SD) for continuous, normally distributed variables and medians (IQD) for non-normally distributed variables.

**Table 2:** Multilevel logistic regression models of neighborhood ethnic diversity and ethnic background on maternal-reported CBCL Total Problems (N = 3076).

		Model 1	Model 2	Model 3
**Individual factor**				
Maternal ethnic background				
*Dutch (ref)*		1.0		1.0
*Non-Dutch* a		2.75 (1.88; 4.04)***		2.64 (1.79; 3.92) ***
	Other European	2.33 (1.39; 3.90)**		2.23 (1.33; 3.76) ***
	Antillean	2.34 (0.92; 6.00)		2.20 (0.86; 5.66)
	Cape Verdean	2.81 (1.30; 6.07)*		2.71 (1.24; 5.90)*
	Moroccan	2.20 (1.12; 4.32)*		2.11 (1.06; 4.19)*
	Surinamese Creole	1.28 (0.43; 3.58)		1.24 (0.41; 3.75)
	Surinamese Hindu	5.19 (2.57; 10.51)***		5.21 (2.57; 10.58)***
	Turkish	3.79 (2.25; 6.41)***		3.76 (2.57; 6.51)***
**Neighborhood factor**				
Neighborhood ethnic diversity				
	Low		1.0	1.0
	Medium		1.47 (0.87; 2.46)	1.13 (0.69; 1.85)
	High		2.03 (1.13; 3.64)*	0.89 (0.51; 1.57)

Models include 60 levels (neighborhoods). Variance (SE) null model 0.39 (0.14); p-value<0.001.

Model 1 is adjusted for child gender, age, maternal age, marital status, parity, maternal educational level, family income Model 2 is adjusted for neighborhood wealth and urbanity level.

Model 3 is the fully-adjusted model.

a Models 1 and 3 repeated with maternal ethnic background categorized as Dutch vs. Non-Dutch.

* p<0.05.

** p<0.01.

*** p<0.001.

Next, we tested whether neighborhood ethnic diversity moderated the association between maternal ethnic background and child behavioral and emotional problems (table 3). When considering interaction particularly in the public health field, it is recommended to present interaction on the additive and the multiplicative scale [29]. Interaction on the additive scale considers absolute risk and is present when the joint effect of two risk factors differs from the sum of the individual risk factors. Interaction on the multiplicative scale considers relative risk and is present when the joint effect of risk factors differs from the product of the effects of the individual factors. Testing for interaction on the additive and the multiplicative scale was conducted in 4 steps as recommended by Knol and VanderWeele. [30]:

**Table 3:** Interaction between maternal ethnic background and neighborhood ethnic diversity on maternal-reported CBCL Total Problems (N = 3076).

		Neighborhood ethnic diversity					
		Low		Medium		High	
		N cases/controls	OR (95%CI)	N cases/controls	OR (95%CI)	N cases/controls	OR (95%CI)
Maternal ethnic background	Dutch	17/852	1.0	36/805	1.71 (0.93; 3.15)	14/425	0.96 (0.43; 2.13)
	Non-Dutch	14/105	5.24 (2.47; 11.14)***	25/227	2.72 (1.35; 5.50)**	90/466	3.03 (1.53; 6.02)**
OR (95% CI) for non-Dutch vs. Dutch within strata of neighborhood ethnic diversity			5.24 (2.47; 11.14)***		1.59 (0.90; 2.80)		3.15 (1.66; 5.99)***
Measure of interaction on additive scale: RERI (95% CI)				−3.22 (−0.70; 0.59) P = 0.097		−1.53 (−5.52; 2.45) P = 0.449	
Measure of interaction on multiplicative scale: Ratio of ORs (95% CI)				0.30 (0.12; 0.76) P = 0.012		0.60 (0.23; 1.58) P = 0.304	

Models include 60 levels (neighborhoods). Variance (SE) null model 0.39 (0.14); p-value<0.001.

OR's are adjusted for child gender, age, maternal age, marital status, parity, maternal educational level, family income, neighborhood wealth and urbanity level.

* p<0.05.

** p<0.01.

*** p<0.001.

1. We presented ORs and CIs for each stratum of neighborhood ethnic diversity and maternal ethnic background with a single reference category (the lowest risk group);
2. We presented ORs with CIs and p-values of the association between maternal ethnic background and behavioral and emotional problems within strata of neighborhood ethnic diversity;
3. We presented measures of interaction on the additive and multiplicative scale with CIs and p-values;
4. We listed the confounders for which the relation of maternal ethnic background and behavioral and emotional problems was adjusted.

As a measure of interaction on the additive scale we presented the Relative Access Risk due to Interaction (RERI) calculated with the following formula [31]:
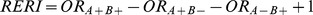
RERI = 0 means no moderation or exact additivity; RERI>0 means positive moderation or more than additivity; RERI<0 means negative moderation or less than additivity.

In order to estimate confidence intervals and p-values around the RERI scores we used the Delta approach [32].

We repeated the interaction analyses with the paternal report of CBCL total problems scale (table S1; supporting information). We additionally repeated the interaction analyses with paternal ethnic background as the determinant and the maternal report of CBCL Total Problems scale as the outcome (table S2; supporting information).

All modeling was conducted in SAS version 9.2 (SAS Institute Inc. 2002–2008).

#### Non-response analysis

Within the Dutch subgroup, mothers who filled in the CBCL at 36 months (n = 2149) were compared with those mothers who did not fill in the questionnaire (n = 452). Data on the CBCL were more often missing in mothers who were single parents (X^2^ 79.4 = 190.1; P<0.001) and lower educated (X^2^ 122.14; P<0.001) but no differences in child birth weight were observed (F = 0.047; P = 0.828) when comparing responders to non-responders. The non-response analyses were repeated in the non-Dutch group and this indicated the same pattern: non-responders were relatively more often lower educated and single parents but children did not have a lower birth weight than responders. A comparison of ethnic minority children included in this study (N = 1649) with children who were excluded due to missing values for maternal ethnicity (N = 226) did not indicate any significant differences in terms of maternal educational level, marital status and child behavioral and emotional problems. We also compared the ethnic minority children included in this study to children who were excluded due to ethnic classification difficulties and small sample sizes (N = 587). We found that the excluded group was higher educated (X2 79.4; P<0.001) than the ethnic minorities that were included. The groups did not differ on marital status and child behavioral and emotional problems. We further compared

## Results

### Characteristics of the study population

Characteristics of the study population are presented in table 1. The mean age of the study participants was 36.6 months (SD 1.3) and 6.5% presented a score above the CBCL total problems cut-off. Mothers of the participants were 31.7 (SD 4.5) years on average, about half was high educated (55.3%) and the family monthly net income was mostly (70.8%) more than 2000 Euros.

### Association of maternal ethnic background and neighborhood ethnic diversity with the CBCL Total Problems score

The associations of maternal ethnic background and neighborhood ethnic diversity with the CBCL Total Problems score are presented in table 2. Variation in behavioral and emotional problems at the neighborhood level (the number of neighborhoods was 60) was significant (i.e. variance (SE) null model 0.39 (0.14); p-value<0.001). In the model adjusted for individual level confounders (model 1), children from ethnic minority groups more often presented behavioral and emotional problems above the cut-off than children classified as Dutch (e.g. Turkish subgroup OR 3.79, 95% CI 2.25; 6.41, P<0.001). In the model adjusted for neighborhood level confounders (model 2), residing in a neighborhood with high ethnic diversity was significantly associated with child behavioral and emotional problems (i.e. OR 2.03, 95% CI 1.13; 3.64, P<0.05) In the fully-adjusted model (model 3) including all neighborhood and individual-level covariates, the associations between the ethnic minority status and child behavioral and emotional problems slightly attenuated but remained significant (e.g. Turkish subgroup OR 3.67, 95% CI 2.13; 6.33, P<0.001). The association between high neighborhood ethnic diversity and child behavioral and emotional problems was no longer significant in the fully-adjusted model (i.e. OR 0.89, 95% CI 0.51; 1.57).

### Moderation by neighborhood ethnic diversity

When comparing medium diversity to low diversity neighborhoods, the interaction with maternal ethnic background trended towards negative on the multiplicative scale (i.e. ratio of ORs 0.30, 95% CI 0.12; 0.76, P = 0.012; see table 3) and on the additive scale (i.e. RERI = −3.23, 95% CI −0.704; 0.59, P = 0.097; see table 3). A similar pattern was found for high diversity versus low diversity neighborhoods however, interactions were not as strong. When stratifying by the three levels of neighborhood ethnic diversity, the results of table 3 show that compared to the Dutch subgroup, the OR for behavioral and emotional problems was significantly increased for ethnic minority children residing in low diversity (i.e. OR 5.24, 95% CI 2.47; 11.14) and high diversity neighborhoods (i.e. OR 3.15, 95% CI 1.66; 5.99) but not in medium diversity neighborhoods (i.e. OR 1.59, 95% CI 0.90; 2.82). In other words, ethnic inequalities in behavioral and emotional problems were greatest in low diversity neighborhoods, slightly smaller in high diversity neighborhoods and smallest in medium diversity neighborhoods. Additional decomposed results show that compared to the Dutch-low diversity group (the lowest risk group), the OR for behavioral and emotional problems was highest for ethnic minority children that reside in low and high diversity neighborhoods (OR 5.24, 95% CI 2.47; 11.14 and OR 3.03, 95% CI 1.53; 6.02, respectfully) and lowest for ethnic minority children that reside in medium diversity neighborhoods (OR 2.72, 95% CI 1.35; 5.50). Hence, there was some indication that the combined effect of being from an ethnic minority group and residing in a medium diverse neighborhood was significantly smaller than the sum of the individual effects of being an ethnic minority and residing in a medium diverse neighborhood.

We repeated the interaction analyses with the paternal report of the CBCL Total Problems as the outcome (N = 2485); this yielded more or less similar results (table S1). Results show that ethnic inequalities were still the greatest in low diversity neighborhoods however; they were smallest in high diversity neighborhoods. We further repeated the interaction analyses with paternal ethnic background instead of maternal ethnic background and the maternal report of the CBCL (N = 2796); this yielded very similar results (table S2).

## Discussion

This study showed that the association between the ethnic minority status and child behavioral and emotional problems may depend on the level of neighborhood ethnic diversity. We found that ethnic inequalities in maternally-reported behavioral and emotional problems were greatest in low diversity neighborhoods, slightly smaller in high diversity neighborhoods and smallest in medium diversity neighborhoods. Additionally, there was some indication that compared to Dutch children in low diversity neighborhoods; minority children presented the least maternally-reported behavioral and emotional problems in medium diversity neighborhoods.

Before discussing the results of this study it is important to address its strengths and limitations. A strength of this study is that it was embedded in a longitudinal birth cohort and as a result elaborate information on ethnic background, child and family characteristics and child behavioral and emotional problems was available. Moreover, this allowed us to select children based on their length of residence in the neighborhood. An additional strength is that we used structural variables to characterize the neighborhood rather than aggregate individual-level variables. Some limitations also need to be discussed. Neighborhood characteristics such as the level of ethnic diversity and ethnic composition are likely to vary within and across countries. Though we do believe that parallels can be drawn between neighborhoods in Rotterdam and urban neighborhoods in other Western European countries, generalizing this study's findings to other settings should be done cautiously. It is also important to note that some families may reside on the border of two zip codes. Although this will only apply to a small group, this could have had a minor influence on the internal validity of the neighborhood level variables. For the interaction analysis, we grouped the children in this study according to maternal ethnic minority status. Although it may have been of interest to look at individual ethnicity (e.g. Turkish), the small sample sizes of the ethnic subgroups did not allow us to do so. Nonetheless, studying the ethnic minority status is of interest as the ethnic minorities share the common characteristic that they do not belong to the ethnic majority and are perceived as culturally different. In turn, ethnic minority groups in the Netherlands often have a marginalized position in society. In this study, some children were excluded due to missing data on ethnic background, ethnic classification difficulties or small sample sizes of some ethnic groups. In a non-response analysis we showed that the excluded children had slightly higher educated mothers than the ethnic minority children included in the study. However, as no differences were observed for other socio-economic characteristics and child behavioral and emotional problems, we do not think that non-response or the exclusion of small ethnic minority groups substantially influenced our findings. We also checked for differences between responders and non-responders on the CBCL at 36 months. Non-responders were more often low educated and single parents than responders. In general, selection towards a higher socio-economic status is a limitation of the Generation R Study [16]. Non-responders however did not differ from responders on child birth weight (an indicator for child health). Due to the cross-sectional nature of our study we cannot distinguish between cause and effect. It is for instance possible that there is social selection into neighborhoods. For instance, families with children that present problematic behavior may move to neighborhoods where there may be more health services (e.g. low diversity neighborhoods) or low SES neighborhoods (e.g. high diversity neighborhoods). It is essential that longitudinal studies are conducted to disentangle cause and effect and that the study is repeated with a larger sample.

In this study, we found that ethnic inequalities in maternally-reported child behavioral and emotional problems were greatest in neighborhoods with a low level of ethnic diversity and smallest in neighborhoods with a medium level of ethnic diversity. Results of other studies conducted in the US and the UK also suggest that the mental health of ethnic minorities may be poorest in homogeneous ‘white’ neighborhoods [9]–[11]. Similar results have also been found in educational settings. For instance, Gottfredson et al. [33] found a positive relationship between the level of ethnic diversity in law schools and educational outcomes such as cognitive openness. However, the interaction with ethnic background was not tested in this study. Further in line with our findings, are the results of a Dutch study which showed that levels of externalizing problems were equal for Dutch and minority students when approximately 2/3 of the population was non-Dutch [9].

There may be several mechanisms that support our findings that (1) ethnic inequalities were greatest in low diversity neighborhoods and smallest in medium diversity neighborhoods and that (2), compared to Dutch children in low diversity neighborhoods, minority children presented the least behavioral and emotional problems in medium diversity neighborhoods. Firstly, ethnically more diverse neighborhoods may also be characterized by higher densities of ethnic groups. Several authors have studied ethnic density effects on physical and mental health outcomes and have noted a protective effect of high ethnic density on the health of minorities as well as the majority group [13], [34]–[36]. Although in our study population ethnic group densities within neighborhoods were never higher than 25% (e.g. for the Turkish) this may still have exerted an influence on child mental health. The mechanisms through which ethnic density is postulated to influence mental health may be similar for ethnic diversity. For instance, racism and discrimination may be more prominently present in neighborhoods with low levels of ethnic diversity which is also true for low levels of ethnic density [11], [34]. Kirkbride et al. (2007) further note that in neighborhoods with a large percentage of majority residents, minority residents are made more aware that they belong to a low status ethnic minority group.

The specific interplay between neighborhood ethnic diversity and racism on mental health has been also studied by Seaton & Yip [5]. They found that the relationship between perceived racism and self-esteem in adolescents was highest in low and high diversity settings and weakest in medium diversity settings. In young children, racism experienced by parents can influence parental affect and attachment styles which in turn can impact behavioral and emotional problems [37].

How can we further explain that ethnic inequalities in maternal reports of child behavioral and emotional problems were smallest in neighborhoods with a medium level of ethnic diversity? One potential explanation is that in neighborhoods with a medium level of ethnic diversity, the positive effects of social connectedness, which is postulated to increase as settings become more ethnically diverse, may enhance the mental health of ethnic minorities [13]. However, when neighborhoods get too diverse, it has been suggested that racial tensions can lead to a breakdown in social cohesion which influences crime rates [38]. Nonetheless, it should be noted that for father reports of child behavioral and emotional problems, ethnic inequalities were found to be smallest in high diversity neighborhoods. Hence, further study, preferably with longitudinal data and a larger sample, into which level of ethnic diversity proves to be the most optimal for the mental health of minority children is required.

## Conclusion

We found that ethnic inequalities in behavioral and emotional problems were greatest in low diversity neighborhoods, slightly smaller in high diversity neighborhoods and smallest in medium diversity neighborhoods. Additionally, compared to Dutch children in low diversity neighborhoods, minority children presented the least behavioral and emotional problems in medium diversity neighborhoods. In order to increase our understanding of the effect of neighborhood diversity on child behavioral and emotional problems in ethnic minorities it is necessary to conduct longitudinal analyses with a larger sample and to gain more insight into the underlying mechanism or mediators. This study suggests that ethnic inequalities in child behavioral and emotional problems may be greatest in ethnically homogeneous neighborhoods.

## Supporting Information

Table S1 **Interaction between maternal ethnic background and neighborhood ethnic diversity on paternal-reported CBCL Total Problems.**
(DOCX)

Table S2 **Interaction between paternal ethnic background and neighborhood ethnic diversity on maternal-reported CBCL Total Problems.**
(DOCX)
