# Supplementary material for: Neighborhood Ethnic Diversity and Behavioral and Emotional Problems in 3 Year Olds: Results from the Generation R Study
Source: PLoS One. 2013 Aug 14;8(8):e70070. doi: 10.1371/journal.pone.0070070 (PMC3743872; doi:10.1371/journal.pone.0070070)
Supplement: Table S1 — Interaction between maternal ethnic background and neighborhood ethnic diversity on paternal-reported CBCL Total Problems. (DOCX) [file pone.0070070.s001.docx]

Table S1 Interaction between maternal ethnic background and neighborhood ethnic diversity on paternal-reported CBCL Total Problems (N=2485)

|  |  | Neighborhood ethnic diversity | | | | | |
| --- | --- | --- | --- | --- | --- | --- | --- |
|  |  | Low | | Medium | | High | |
|  |  | N cases/controls | OR (95%CI) | N cases/controls | OR (95%CI) | N cases/controls | OR (95%CI) |
| Maternal ethnic background | Dutch | 34/735 | 1.0 | 42/673 | 1.19  (0.71; 1.97) | 26/333 | 1.13  (0.60; 2.11) |
|  | Non-Dutch | 12/86 | 2.74  (1.35; 5.55)** | 26/148 | 2.69  (1.47; 4.92)** | 50/320 | 1.46  (0.77; 2.75) |
| OR (95% CI) for non-Dutch vs. Dutch within strata of neighborhood ethnic diversity |  |  | 2.74  (1.35; 5.55)** |  | 2.27  (1.31; 3.91)** |  | 1.29  (0.72; 2.30) |
| Measure of interaction on additive scale RERI (95% CI) | | | | -0.24 (-1.97; 1.49) P=0.786 | | -1.41 (-3.47; 0.64) P=0.179 | |
| Measure of interaction on multiplicative scale Ratio of ORs (95% CI) | | | | 0.83 (0.34; 1.99) P=0.672 | | 0.47 (0.19; 1.15) P=0.099 | |

Models include 60 levels (neighborhoods). Variance (SE) null model 0.15 (0.10); p-value <0.01

OR’s are adjusted for child gender, age, maternal age, marital status, parity, maternal educational level, family income, neighborhood wealth and urbanity level

* p<0.05 ** p<0.01 *** p<0.001
