# Supplementary material for: Neighborhood Ethnic Diversity and Behavioral and Emotional Problems in 3 Year Olds: Results from the Generation R Study
Source: PLoS One. 2013 Aug 14;8(8):e70070. doi: 10.1371/journal.pone.0070070 (PMC3743872; doi:10.1371/journal.pone.0070070)
Supplement: Table S2 — Interaction between paternal ethnic background and neighborhood ethnic diversity on maternal-reported CBCL Total Problems. (DOCX) [file pone.0070070.s002.docx]

Table S2 Interaction between paternal ethnic background and neighborhood ethnic diversity on maternal-reported CBCL Total Problems (N=2796)

|  |  | Neighborhood ethnic diversity | | | | | |
| --- | --- | --- | --- | --- | --- | --- | --- |
|  |  | Low | | Medium | | High | |
|  |  | N cases/controls | OR (95%CI) | N cases/controls | OR (95%CI) | N cases/controls | OR (95%CI) |
| Maternal ethnic background | Dutch | 74/415 | 1.0 | 18/201 | 1.40  (0.77; 2.56) | 10/109 | 0.80  (0.36; 1.77) |
|  | Non-Dutch |  | 2.84  (1.27; 6.33)* |  | 1.55  (0.73; 3.30) |  | 1.89  (0.93; 3.84) |
| OR (95% CI) for non-Dutch vs. Dutch within strata of neighborhood ethnic diversity |  | 14/381 | 2.84  (1.27; 6.33)* | 34/739 | 1.10  (0.58; 1.04) | 20/781 | 2.37  (1.21; 2.26)* |
| Measure of interaction on additive scale RERI (95% CI) | | | | -1.68 (-3.93; 0.56) P=0.141 | | -0.75 (-3.17; 1.68) P=0.547 | |
| Measure of interaction on multiplicative scale Ratio of ORs (95% CI) | | | | 0.39 (0.14; 1.07) P=0.068 | | 0.84 (0.30; 2.31) P=0.729 | |

Models include 60 levels (neighborhoods). Variance (SE) null model 0.39 (0.14); p-value <0.001

OR’s are adjusted for child gender, age, maternal age, marital status, parity, maternal educational level, family income, neighborhood wealth and urbanity level

* p<0.05 ** p<0.01 *** p<0.001
